# Twelve-Year Follow-Up of Laser Frenectomy during Early Mixed Dentition

**DOI:** 10.1155/2023/5525534

**Published:** 2023-12-29

**Authors:** Elhadi Mohieldin Awooda

**Affiliations:** Department of Restorative Dentistry and Laser Dentistry, Napata College, Khartoum, Sudan

## Abstract

Median maxillary labial frenum (MMLF) is one of the oral freni, found on the inner side of the centre of the upper lip. Maxillary midline diastema (MMD) is commonly associated with highly attached frenum, and frenectomy during early mixed dentition is controversial. A 6-year-old boy came with a chief complaint of unpleasant spacing between the two upper front teeth. A median maxillary high attached labial frenum with midline spacing of 5 mm was diagnosed. A consensus was made with the child's father to remove the frenum by laser. Diode laser frenectomy was done, and subsequent follow-up for 12 years revealed no relapse and complete closure of the diastema. Frenectomy during early mixed dentition could be a suitable option for the closure of midline diastema.

## 1. Introduction

Median maxillary labial frenum (MMLF) is one of the oral freni, found on the inner side of the centre of the upper lip; it connects to the midline of the attached gingiva between the two central incisors [1]. Depending upon the extension of attachment of fibers, freni have been classified as mucosal, gingival, papillary, and papilla penetrating [2]. A new classification was created named Stanford classification which is based on the appearance of the frenum and classifies it into three types [3]. Aberrant frenum often causes problems such as loss of papilla, recession, diastema, difficulty in brushing, and alignment of the teeth [4]. Maxillary midline diastema (MMD) is commonly associated with highly attached frenum, and its occurrences are approximately 98% of 6-year-olds, 49% of 11-year-olds, and 7% of 12–18-year-olds [5]. It is potentially leading to facial-cervical caries as well as initiation and progression of gingival/periodontal disease [6].

Controversy arises on clear indication and timing of the treatment of midline diastema due to high attached frenum [7]. There is a consensus between “paediatric dentists and orthodontists that frenectomy should not be performed before the permanent canines erupt and then following the closure of the space orthodontically” [8]. On the other hand, a child's psychological well-being, parents' worries, unpredictable later outcomes of its closure, and costly multidisciplinary approaches may necessitate early intervention for its treatment in early childhood during the primary or mixed dentition. Frenectomy can be accomplished either by the conventional scalpel technique, by electrosurgery, or by using laser technology [9]. This case presents a diode laser frenectomy with a 12-year follow-up to a six-year-old boy complaining of an unpleasant appearance due to midline diastema.

## 2. Case Presentation

### 2.1. Patient Information

A 6-year-old boy came to the dental clinic at the laser institute (Sudan University of Sciences and Technology), complaining of spacing between the two upper front primary teeth, where his peers at school bullied him for his appearance. His parents noticed the spacing of the primary teeth and worried about the fate of the permanent ones as the diastema has occurred since the eruption of his primary teeth. The child looked well, not pale, or jaundiced, and no abnormalities were mentioned in his past medical history.

### 2.2. Clinical Findings

**Figure 1:**
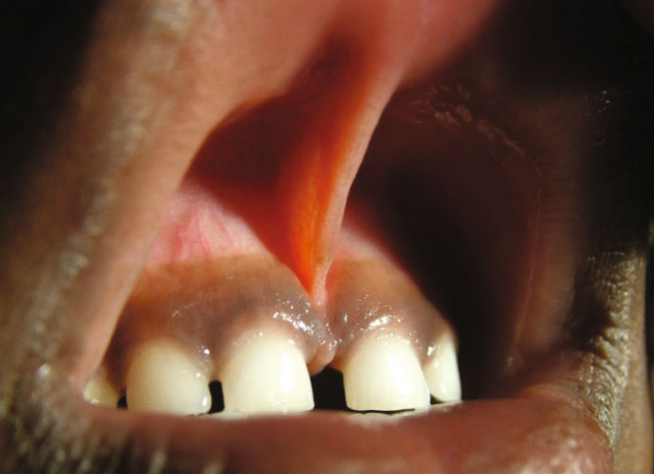
Preoperative photo shows high attached midline frenum with 5 mm spacing between the two central incisors and 1.5 mm between laterals and canines.

Clinical intraoral examination revealed the presence of midline diastema of 5 mm width associated with high attachment of the papillary type of frenum (Figure 1). In addition, spaces of 1-2 mm were found between lateral incisors and canines on both sides. Gingival pigmentation was seen with normal color and texture, and no periodontal pockets were detected. Examination of the teeth and other soft tissues revealed no abnormalities.

### 2.3. Diagnostic Assessment and Treatment Planning

The case was diagnosed clinically (no other diagnostic aids used such as X-rays) as high attachment of papillary type of frenum and maxillary labial midline diastema of a 5 mm width. Treatment planning was laser frenectomy, and this plan was based on the concept of “evidence-based dentistry” as follows: my previous experience in laser frenectomy [10], similar cases treated by lasers retrieved from the literature, and parents' preference for frenectomy by laser [11]. The mechanism of how laser cut the tissue was explained to the child's father with its most positive outcome of no pain, no bleeding, and with the use of a few drops of local anesthesia. The father opted for the treatment by laser, and he signed an informed written consent for both, frenectomy by laser and publication of the case.

### 2.4. Therapeutic Intervention

**Figure 2:**
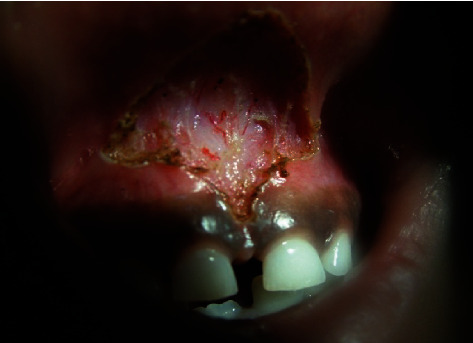
Immediately postoperatively shows excised frenum by diode laser 810 nm without bleeding.

A topical anesthetic spray was applied before the local anesthesia administration. The surgical area was anesthetized with a few drops of lignocaine with 1 : 80,000 adrenaline (LOX 2% Adrenaline). The surgery was performed by the diode laser 810 nm (Oralia Company, Germany), at a power setting of 2 watts and a size 400-micron fiberglass tip in continuous wave mode. The laser fiber contacted the tissues and moved vertically and laterally to the frenum which initially cut off the mucosa continuity, thereby allowing performing a deeper cut of the frenum in a horizontal dimension, and its apex was excised from the base. The whole procedure was completed within 10-minute duration, with no bleeding, pain, or discomfort, and the cut part was left without suturing and no periodontal pack applied (Figure 2). The child had no pain postoperatively and was discharged uneventfully with no analgesia or antibiotic prescribed. The father was instructed to close supervision of his child's oral hygiene to avoid mechanical trauma by tooth brush and acidic foods and drinks during the first week. 

### 2.5. Follow-Up and Outcome

**Figure 3:**
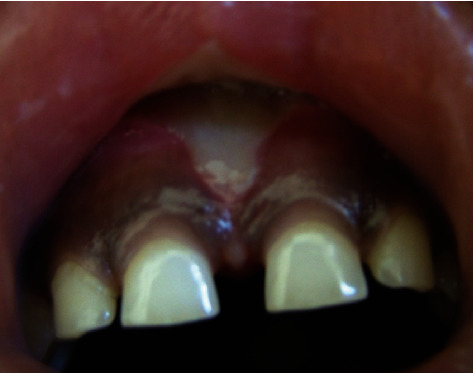
Three days postoperatively of laser frenectomy shows whitish fibrin tissue formation indicating an early healing process.

The child was recalled three days postoperatively, with no history of pain or discomfort, and the healing site showed a fibrin tissue formation (Figure 3).

**Figure 4:**
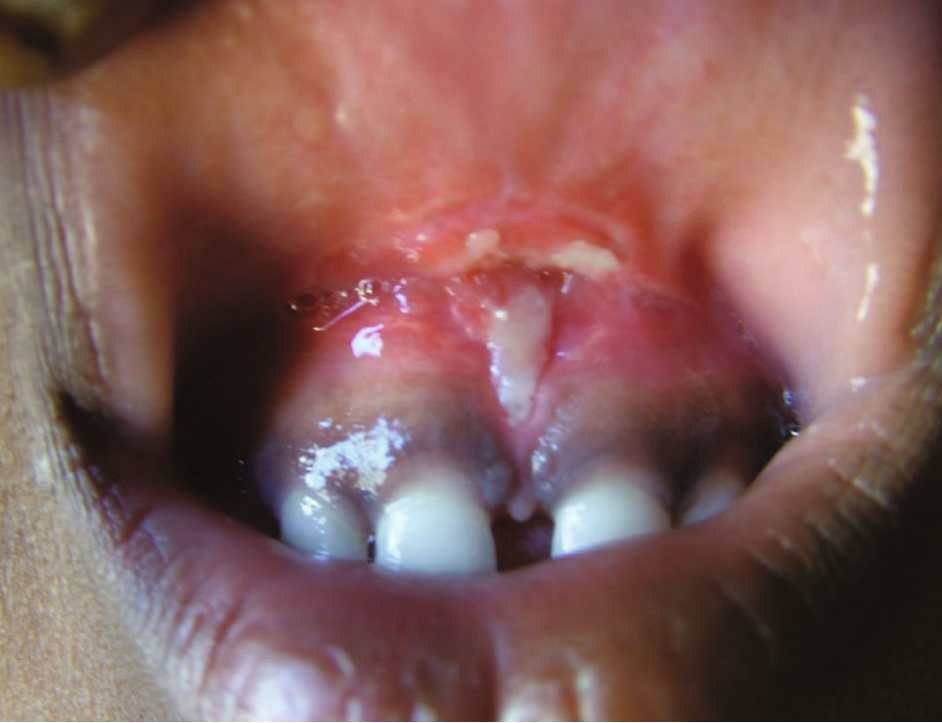
Ten days after laser frenectomy shows signs of getting complete healing process.

**Figure 5:**
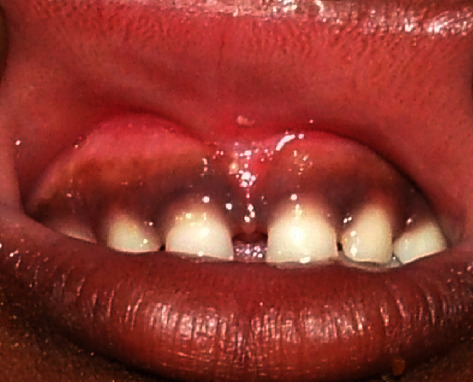
After one month of diode laser frenectomy, complete healing of tissues with lowered attached frenum.

**Figure 6:**
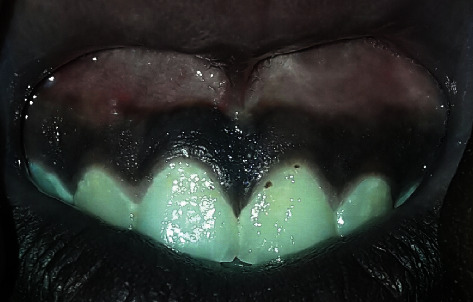
8 years postoperatively shows normal contact between the teeth and the normal position of the maxillary medium labial frenum.

**Figure 7:**
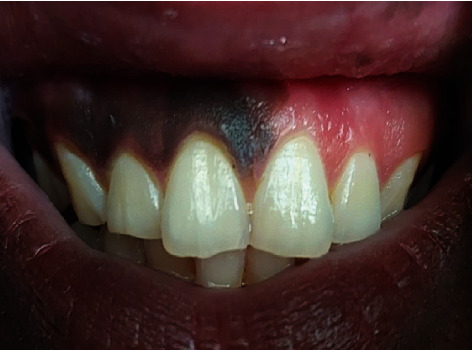
12-year postoperatively shows normal alignment of permanent anterior teeth with self-depigmented gingiva on the left side.

The child was called for follow-up in 10 days (Figure 4) and then followed regularly (Figures 5 and 6) and up to 12-year duration (Figure 7).

Follow-up clinically and photographically has revealed the closure of the midline diastema with no spacing on the permanent dentition.

### 2.6. Patient Perspective

The child's age now is 17 years old, and he does not remember his diastema or the whole previous surgical procedure. His father confirmed no restorative/orthodontic treatments or further consultations, and the diastema closed on its own.

## 3. Discussion

Aesthetic demands become one of the most important concerns in seeking dental care for achieving a better look, regardless of patient age or sex [3]. Midline diastema could persist in adulthood and may be considered an unaesthetic or malocclusion problem [7]. High attached frenum interferes with proper oral hygiene measures, potentially leading to dental caries and gingival/periodontal disease [3, 5]. Several treatment modalities were proposed, ranging from the classic frenectomy or orthodontic treatment to more radical procedures of subapical osteotomies, corticotomies, septotomies, and reverse-bevel gingivectomy [1]. Physiological closure of the midline diastema in permanent dentition is inevitability among a majority of cases [3, 12]. Also, it could not be predicted among whom it may persist. Early surgical intervention may eliminate the need for future orthodontic/restorative correction during permanent dentition [13]. It also boosts patient self-esteem, along with cost-effectiveness, besides the advantages of being painless and bloodless, when the procedure is performed by laser [4].

Based on the statement that maxillary frena are dynamic structures subjected to variations during different stages of human growth and development [3, 14], there is general agreement that frenectomy should not be performed before the permanent canines erupt and the closure of the space should be orthodontically and/or restoratively [8, 12, 15]. However, our successful experience of this case of laser frenectomy during the early mixed dentition contradicted the above-mentioned statement as the follow-up for 12 years has revealed the eruption of the permanent teeth in normal occlusion. Others suggested that diastema is rarely closed spontaneously during further development, if it is more than 2 mm or if the attachment exerts a traumatic force on the gingiva [3, 12, 15, 16]. After a long follow-up and successful outcome of the treatment of this case, the time chosen for the frenectomy procedure showed magnificent excellence in the alignment of permanent teeth, and it agrees with Richard et al. that it does not form scar tissue that prevents diastema closure [17].

The conventional surgical technique for frenectomy involves the excision of the frenum using a scalpel through different techniques, like Miller's, V-Y plasty, and Z-plasty [9]. Surgical excision of the frenum has drawbacks such as patient fear and anxiety, especially for children, besides intraoperative bleeding, postoperative hematoma, pain, and discomfort [18]. Alternatively, the laser is becoming the standard of care for many surgical procedures because of its improved precision, visualization, reduced hemorrhage, and less postoperative complications thereby enhancing the patient's comfort, especially for young children.

Diode laser when compared to other lasers such as Nd:YAG is known for its high absorbance by pigmented tissues with hemoglobin or melanin; therefore, it is commonly used for frenectomy procedures [4, 19]. Laser provides better postoperative patient perceptions than the conventional technique [19]. Different wavelengths can be used, but the principal concept to remember for all wavelengths is that the minimum effective energy must be used because the lower the energy applied, the less damage to the targeted tissue and the faster the healing process [20, 21]. In this case, laser excision seems more convenient because of no bleeding, no need for suture, and no unaesthetic sequelae [22–24]. The laser cut a huge wound and no suturing; so, few studies such as the one done by Komori et al. advise the use of prophylaxis antibiotics after laser surgery [25]. But in agreement with others, there is no need for antibiotic coverage [26, 27], and this is because the laser beam ensures that the incised tissue is sterilized during the surgical process. Thus, the risk of postoperative infection is reduced [28].

After 12 years of follow-up, diode laser frenectomy was effective and the surgical area healed uneventfully, with the frenum repositioned in its normal anatomical contour, and the diastema closed permanently. The patient did not have any postoperative complications, thus demonstrating the laser's effectiveness.

## 4. Limitation

I regret the suboptimal quality of the photographs taken, and the different color contrast throughout the too-long follow-up as the pictures were taken by different cameras of different resolutions. Also, the issue of child gingival hyperpigmentation and depigmentation is not discussed in this case report as I narrowed my description in the variables of high attached labial frenum, midline diastema, and early laser frenectomy with long follow-up.

## 5. Conclusion

Surgical excision of frenum during early childhood by using a laser reduces surgical time, no suture placement, no postoperative pain or discomfort, absence of scarring, and no recurrence, and it was closed without the need for orthodontic/restorative intervention.
